# Supplementary material for: Hypoxia-Reoxygenation Impairs Autophagy-Lysosomal Machinery in Primary Human Trophoblasts Mimicking Placental Pathology of Early-Onset Preeclampsia
Source: Int J Mol Sci. 2022 May 18;23(10):5644. doi: 10.3390/ijms23105644 (PMC9147570; doi:10.3390/ijms23105644)
Supplement: Supplementary file 1 [file ijms-23-05644-s001.zip › ijms-1705765-supplementary.pdf]

International Journal of
Molecular Sciences

MDPI

Supplemental Table

Hypoxia–Reoxygenation Impairs Autophagy–Lysosomal Machinery in Primary Human Trophoblasts Mimicking Placental Pathology of Early-Onset Preeclampsia

Shibin Cheng [1,*], Zheping Huang [1], Sukanta Jash [1], Kathleen Wu [1], Shigeru Saito [2], Akitoshi Nakashima [2] and Surendra Sharma [1]

[1] Department of Pediatrics, Women &Infants Hospital of Rhode Island, Warren Alpert Medical School of Brown University, Providence, RI 02905, USA; zhuang@wihri.org (Z.H.); sjash@wihri.org (S.J.); kathleen_wu@brown.edu (K.W.); ssharma@wihri.org (S.S.)
[2] Department of Obstetrics and Gynecology, Faculty of Medicine, University of Toyama, Toyama, Japan; s30saito@med.u-toyama.ac.jp (S.S.); akinaka@med.u-toyama.ac.jp (A.N.)
* Correspondence: shibin_cheng@brown.edu; Tel.: 401-430-8007

Table S1. Demographic and clinical characteristics of patients.

Variable	Early onset preeclampsia (*n* = 7)	Gestational age-matched control (*n* = 7)	*p*-value
Age (years)	29.8 (7.1)	23.7 (5.9)	†† 0.14[1]
Race			
White	3 (42.9%)	3 (42.9%)	†† 0.84[2]
Black	1 (14.3%)	1 (14.3%)	
Hispanic	3 (42.9%)	3 (42.9.5%)	
Other	0	0	
BMI (kg/m^2)	31.4 (6.5)	32.9 (6.3)	†† 0.87[1]
Gestational age at delivery (weeks)	30.8 (1.7)	31.5 (2.5)	†† 0.06[3]
Maternal temperature (°C)	36.9 (0.13)	36.8 (0.21)	†† 0.36[1]
Maximum systolic blood pressure (mmHg)	180.2 (13.7)	119.3 (11.5)	††<0.001[3]
Maximum diastolic blood pressure (mmHg)	113.1 (6.3)	73.5 (3.8)	††<0.001[3]
Mode of delivery			
Vaginal	2 (28.6%)	4 (57.1%)	†† 0.62[2]
Cesarean Section	5 (71.4%)	3 (42.9%)	
Maternal hemoglobin (g/dl)	10.4 (0.9)	10.5 (1.6)	†† 0.81[1]
Maternal platelets (×10^3/μl)	105.1 (72.5)	179.5 (50.5)	†† 0.02[1]
AST (U/L)	226.3 (304.4)	11 – 30*	N/A
Serum creatinine (mg/dl)	0.98 (0.27)	0.5 – 1.1*	N/A
Urine protein: creatinine	5.0 (6.6)	< 0.3*	N/A
Birth weight (grams)	1334 (129)	2133 (382)	†† < 0.001[1]

Data presented as Mean (standard deviation) for continuous variables.
Data presented as n (%) for categorical variables.
*AST, serum creatinine, and urine protein:creatinine not measured in control subjects and presented as normal ranges.
[1]t-test.
[2] Fisher's exact test.
[3] Wilcoxon rank-sum †† early-onset preeclampsia with severe features versus gestational age-matched controls.
